# Referred Pain Manifestation and Its Impact on Patients With Temporomandibular Disorder

**DOI:** 10.1111/odi.70021

**Published:** 2025-07-21

**Authors:** Beatriz Amaral de Lima‐Netto, Paulo César Rodrigues Conti, Rafaela Stocker Salbego, Matheus Herreira‐Ferreira, Yuri Martins Costa, Leonardo Rigoldi Bonjardim

**Affiliations:** ^1^ Department of Biological Sciences, Bauru School of Dentistry University of São Paulo Bauru Brazil; ^2^ Department of Prosthodontics and Periodontology, Bauru School of Dentistry University of São Paulo Bauru Brazil; ^3^ Department of Biosciences, Piracicaba School of Dentistry University of Campinas Piracicaba Brazil

**Keywords:** biopsychosocial, chronic pain, palpation, referred pain, temporomandibular disorder

## Abstract

**Objective:**

To investigate whether a higher number of sites eliciting referred pain upon palpation is associated with greater impairment in biopsychosocial aspects in TMD patients and identify variables that predict increased referred pain.

**Methods:**

This cross‐sectional study analyzed data from 77 women with myalgia, assessing the number of sites eliciting referred pain upon palpation in relation to clinical (duration, frequency, intensity, painful body sites, pain‐related disability), psychological (stress, anxiety, depression, catastrophizing, sleep quality), and psychophysical variables (mechanical and pressure pain thresholds, temporal summation and conditioned pain modulation).

**Results:**

Spearman's correlation revealed a significant correlation between the number of sites eliciting referred pain upon palpation in the orofacial region and both pain intensity and frequency (*p* < 0.001), as well as with the number of painful body sites (*p* = 0.009). Negative binomial regression indicated that each additional day of pain in the past week and each additional painful body site were predictors of increases of 10% and 4%, respectively, in the number of sites eliciting referred pain upon palpation.

**Conclusions:**

These findings highlight the utility of assessing referred pain sites as a complementary indicator of severity in clinical pain parameters, providing a practical and effective tool for a more comprehensive evaluation of patients.

## 1 Introduction

Referred pain is a clinical feature manifested in several painful musculoskeletal conditions (Kosek and Januszewska 2008; Alonso‐Blanco et al. 2012; Bron et al. 2011). It is defined as a sensation perceived outside the anatomical boundaries of the region where the nociceptive stimulus originates (Graven‐Nielsen 2006). The prevalence of referred pain can be considerable, reaching up to 60.7% among individuals with temporomandibular disorders (TMD) (Alketbi and Talaat 2022). The relevance of referred pain is widely acknowledged, being included in the Diagnostic Criteria for Temporomandibular Disorders (DC/TMD) (Schiffman et al. 2014) and in the International Classification of Orofacial Pain (ICOP) (*Cephalalgia* 2020).

Although the underlying neuronal mechanism is not fully understood, widely accepted theories involve neuronal convergence and the expansion of receptive fields (Graven‐Nielsen 2006; Coghill et al. 1991). This phenomenon can be explained by the cross‐talk of information from primary peripheral neurons to a common central secondary neuron. From this anatomical distribution, an additional recruitment of wide dynamic range (WDR) neurons, which are not specific to nociceptive stimuli, appears to activate silent synapses and expand their receptive fields (Hoheisel et al. 1993). This process would result in the clinical perception of sensation in areas beyond the origin of the nociceptive stimulus (Graven‐Nielsen 2006).

It is believed that this processing is facilitated by both peripheral and central components (Graven‐Nielsen 2006; Sago et al. 2023), with hyperexcitability of the system in response to nociceptive stimuli, reduced pain thresholds (Hong et al. 1996), and deficiency in the endogenous pain modulation system (Sago et al. 2023; Graven‐Nielsen et al. 2000). From this perspective, referred pain has been considered an indicator of greater complexity within the overall context of patients with chronic pain (Graven‐Nielsen 2006; Arendt‐Nielsen et al. 2000).

The pathophysiological significance of this characteristic, as well as its therapeutic implications, remains unclear. The current categorization (Schiffman et al. 2014) suggests a hierarchy between local myalgia, myofascial pain with spreading, and myofascial referred pain, but does not consider the potential presence of the same disorder manifested at different stages (Michelotti et al. 2016). Further studies have been proposed to improve diagnostic criteria and clarify whether referred pain is merely an epiphenomenon or if it possesses clinical relevance for classification and treatment guidance (Michelotti et al. 2016).

So far, few studies in the scientific literature have addressed this specific topic (Winocur‐Arias et al. 2022; Barjandi et al. 2021; Varun et al. 2023; Lovgren et al. 2022). Some clinical parameters, such as pain intensity (Winocur‐Arias et al. 2022) and the spread of painful areas across the body (Lovgren et al. 2022), have shown weak associations with the presence of referred pain. In contrast, severe psychosocial parameters, assessed by the “Axis II” of the DC/TMD, have shown stronger associations (Winocur‐Arias et al. 2022; Barjandi et al. 2021; Varun et al. 2023).

In this context, uncertainty remains regarding whether referred pain can be considered a reliable indicator of severity, particularly since the diagnosis requires only one referred palpation site, either in the masseter or temporalis muscle (Schiffman et al. 2014). It is also unclear whether an increase in referred pain during the physical examination, as currently assessed by the DC/TMD, reflects an additional biopsychosocial burden in patients with TMD. Moreover, although the ICOP (*Cephalalgia* 2020) recognizes referred pain elicited by the temporomandibular joint as a diagnostic criterion, it remains uncertain whether this manifestation has distinct clinical implications, considering the different underlying pathophysiological pain mechanisms of these structures (Cairns 2010).

Therefore, the primary objective of this study was to determine whether a higher number of sites eliciting referred pain upon palpation in the orofacial region is associated with additional negative impact on clinical, psychosocial, and psychophysical parameters in individuals with TMD. The secondary objective was to identify which variables within the biopsychosocial framework significantly predict the number of sites eliciting referred pain during physical examination.

## 2 Methodology

### 2.1 Study Design

This cross‐sectional study was based on a secondary data analysis, which refers to the use of data originally collected for a different primary research purpose. The study was approved by the Research Ethics Committee of the Bauru School of Dentistry at the University of São Paulo (CAAE 63551122.7.0000.5417). All individuals in the original investigation provided written informed consent. The primary study aimed to evaluate endogenous pain modulation using duloxetine in patients with painful TMD.

The data set comprised 77 female participants, all diagnosed with myalgia based on the DC/TMD criteria, with the potential co‐diagnosis of arthralgia. The primary study's inclusion criteria for women with TMD included: being over 18 years old; receiving a clinical diagnosis of myalgia or myofascial pain, either with or without accompanying arthralgia of the TMJ and/or headache attributed to TMD; and experiencing pain for a duration of at least 90 days. Exclusion criteria encompassed uncontrolled systemic illnesses such as diabetes, hypertension, or endocrine dysfunctions; epilepsy; cardiovascular, hepatic, or renal disease; neuropathies; psychiatric conditions including bipolar disorder; substance abuse within the previous year; suicidal ideation; use of serotonin‐norepinephrine reuptake inhibitors (SNRIs) within the past 12 months or monoamine oxidase inhibitors (MAOIs) within 14 days prior to participation; pregnancy or breastfeeding; and any TMD‐related treatment in the preceding 3 months.

The evaluation of all participants was performed by the same experienced specialist in TMD. For data analysis and classification, item 9, “Pain on palpation”, from the DC/TMD (Schiffman et al. 2014) was employed. The physical examination followed the DC/TMD guidelines, asking whether the pain location remained under the examiner's fingertip or radiated elsewhere during palpation with 1 kg of digital pressure for 5 s on masseter and temporalis muscles bilaterally. For the temporomandibular joints, the same procedure was performed, but with calibrated pressure of 0.5 kg on the lateral pole and 1 kg around the lateral pole. The number of sites eliciting referred pain upon palpation was recorded in the temporal (posterior, middle, and anterior) and masseter muscles (origin, body, and insertion), as well as the temporomandibular joint (lateral pole and around lateral pole), bilaterally, resulting in a scale ranging from 0 to 16 points. Palpations that elicited pain with spreading were excluded from this count and categorized as “0”, indicating no referred pain upon palpation.

All palpation sites that exhibited referral—defined as a sensation perceived outside the boundaries of the stimulus's origin—were reported by the patients as familiar pain.

### 2.2 Clinical Parameters

In this study, the participants were instructed to fill out a medical history form indicating the self‐reported pain intensity, duration of pain since its onset (in months), and the frequency of pain days in the past week. Additionally, variables related to the number of painful body sites, the chronic pain intensity, and pain‐related disability were also assessed, as detailed below.

#### 2.2.1 Pain Drawing

The distribution of painful body sites was evaluated using the Pain Drawing tool from the DC/TMD protocol (Schiffman et al. 2014). Participants were instructed to indicate, on a body diagram, all areas where they had experienced pain in the preceding month. The total number of marked sites—ranging from 0 to 45—was counted by the researcher, with higher scores reflecting greater widespread pain (Margolis et al. 1986). It is noteworthy that this measure reflects the general distribution of pain across the body and does not represent referred pain, which was assessed separately through standardized palpation procedures in the trigeminal region.

#### 2.2.2 Graded Chronic Pain Scale (GCPS)

##### 2.2.2.1 Chronic Pain Intensity (CPI)

Pain intensity over the past month was measured using the Graded Chronic Pain Scale (Von Korff et al. 1992). The Chronic Pain Intensity (CPI) score was calculated by taking the average of three pain ratings (current pain, worst pain, and average pain in the past month), then multiplying by 10. Ratings ranged from 0 (no pain) to 10 (worst imaginable pain). Final scores were rounded to the nearest whole number, with decimals below 0.5 rounded down and those 0.5 or higher rounded up.

##### 2.2.2.2 Pain‐Related Disability

Functional disability was assessed using a scale from 0 to 6. This score was based on the average of three interference ratings—daily activities, work, and social life. Each rating ranged from 0 (no interference) to 10 (unable to perform any activity) and was multiplied by 10. Additionally, the number of days the participant was unable to perform usual activities due to pain in the past month was added to the total score (Von Korff et al. 1992).

### 2.3 Psychosocial Parameters

Psychological and sleep‐quality aspects were measured using standardized self‐report questionnaires. The assessments included perceived stress, anxiety and depression symptoms, pain catastrophizing, and sleep quality, as outlined below. All instruments were administered in their validated Portuguese versions.

#### 2.3.1 Perceived Stress Scale (PSS) (Cohen et al. 1983)

This 14‐item questionnaire assesses the extent to which individuals perceive life events as unpredictable, uncontrollable, and overwhelming. The total score is the sum of all responses, ranging from 0 to 56 points (Cohen et al. 1983; Reis et al. 2010).

#### 2.3.2 Hospital Anxiety and Depression Scale (HADS) (Zigmond and Snaith 1983)

The HADS is a 14multiple‐choice questionnaire divided into two subscales: one for anxiety and one for depression, with seven items each. Scores range from 0 to 21. Higher scores indicate more pronounced symptoms of anxiety and/or depression (Zigmond and Snaith 1983; Castro et al. 2006).

#### 2.3.3 Pain Catastrophizing Scale (PCS) (Sullivan et al. 1995)

This instrument evaluates the tendency to engage in negative and exaggerated thoughts about pain. It includes 13 items rated on a 0–5 scale, where 0 = “almost never” and 5 = “almost always.” The total score ranges from 0 to 52, with higher scores indicating more severe catastrophizing (Sullivan et al. 1995; Sehn et al. 2012).

#### 2.3.4 Pittsburgh Sleep Quality Index (PSQI) (Buysse et al. 1989)

The PSQI contains 19 items assessing various dimensions of sleep, including sleep duration, latency, frequency, and intensity of disturbances. Higher total scores indicate poorer subjective sleep quality (Buysse et al. 1989; Bertolazi et al. 2011).

### 2.4 Psychophysical Parameters

To assess pain sensitivity, quantitative sensory tests were performed, including mechanical pain threshold (MPT), wind‐up ratio (WUR), pressure pain threshold (PPT), and conditioned pain modulation (CPM). Assessments were conducted in both trigeminal and extra‐trigeminal regions, on the most painful side of the masseter muscle and on the ipsilateral hand.

#### 2.4.1 Mechanical Pain Threshold (MPT)

Superficial mechanical pain sensitivity was measured using nylon Von Frey filaments (Touch Test – North Coast), which apply forces ranging from 0.008 to 300 g/mm^2^. Filaments were applied perpendicularly to the masseter and thenar regions. Filaments were presented in ascending order until a painful sensation was reported (positive result), and then in descending order until no pain was perceived (negative result). This cycle continued until five positive and five negative thresholds were recorded. The geometric mean of these values was used as the final MPT score (Rolke et al. 2006).

#### 2.4.2 Wind‐Up Ratio (WUR)

To assess temporal summation of pain, a low‐caliber Von Frey filament (Touch Test – North Coast) was used to produce a mild painful sensation. It was applied a single stimulus followed by a series of 10 repetitive stimuli (one per second) to the masseter and thenar region, until a slight curvature was observed. Participants rated pain intensity (0–100) after both the single and repeated applications. This procedure was repeated three times at each site. The value was calculated by dividing the mean pain rating of the repeated stimuli by the mean rating of the single stimuli (Rolke et al. 2006).

#### 2.4.3 Pressure Pain Threshold (PPT)

Deep tissue pain sensitivity was assessed using a digital pressure algometer (Medoc AlgoMed, Medoc Ltd., Israel) with a 1 cm^2^ flat probe. Pressure was gradually increased at a rate of approximately 0.5 kg/cm^2^ per second. Participants pressed a button as soon as the pressure became slightly painful. The final value was the average of three consecutive trials (Rolke et al. 2006).

#### 2.4.4 Conditioned Pain Modulation (CPM)

To evaluate endogenous pain inhibition, the sequential CPM protocol was applied. PPT values were measured in the masseter and thenar muscles both before and after the conditioning stimulus. The conditioning involved immersing the contralateral hand in cold water (10°C–16°C) for 60 s, with the temperature individually adjusted according to each participant's tolerance (Granot et al. 2008; Nir et al. 2011). The modulation effect was calculated as the absolute difference in PPT values before and after the cold‐water stimulus (Yarnitsky et al. 2010).

### 2.5 Statistical Analysis

The data was analyzed using Jamovi 2.4.14 software. Descriptive statistics were used to characterize the sample. The normality of variables was assessed using the Shapiro–Wilk test. For all tests, differences were considered statistically significant when *p* < 0.05.

Initially, a Spearman correlation analysis was performed since the variable “Number of sites eliciting referred pain upon palpation” did not present a normal distribution (*p* < 0.001). In a second step, after identifying the variables with statistically significant correlations, a regression analysis was conducted to assess whether these variables could predict the outcome, number of sites eliciting referred pain upon palpation, in the orofacial region.

Due to the non‐normal distribution of the data and the unsuitability of multiple linear regression, a quasi‐Poisson regression model with robust variance was initially considered. Given the presence of moderate overdispersion—characterized by the variance exceeding the mean of the dependent variable—the negative binomial regression model was selected as a more appropriate analytical approach. This model yielded more reliable and robust parameter estimates compared to the quasi‐Poisson regression.

## 3 Results

### 3.1 Sample Characterization

The study analyzed cross‐sectional data from 77 women diagnosed with painful TMD. The distribution of muscular diagnoses was as follows: 18.18% (14 of 77) with local myalgia, 15.59% (12 of 77) with myofascial pain with spreading, and 66.23% (51 of 77) with referred myofascial pain. An associated diagnosis of arthralgia was present in 84.4% (65 of 77) of the sample. Among the patients with arthralgia (65), 69.23% (45) reported referred arthralgia upon palpation.

**FIGURE 1:**
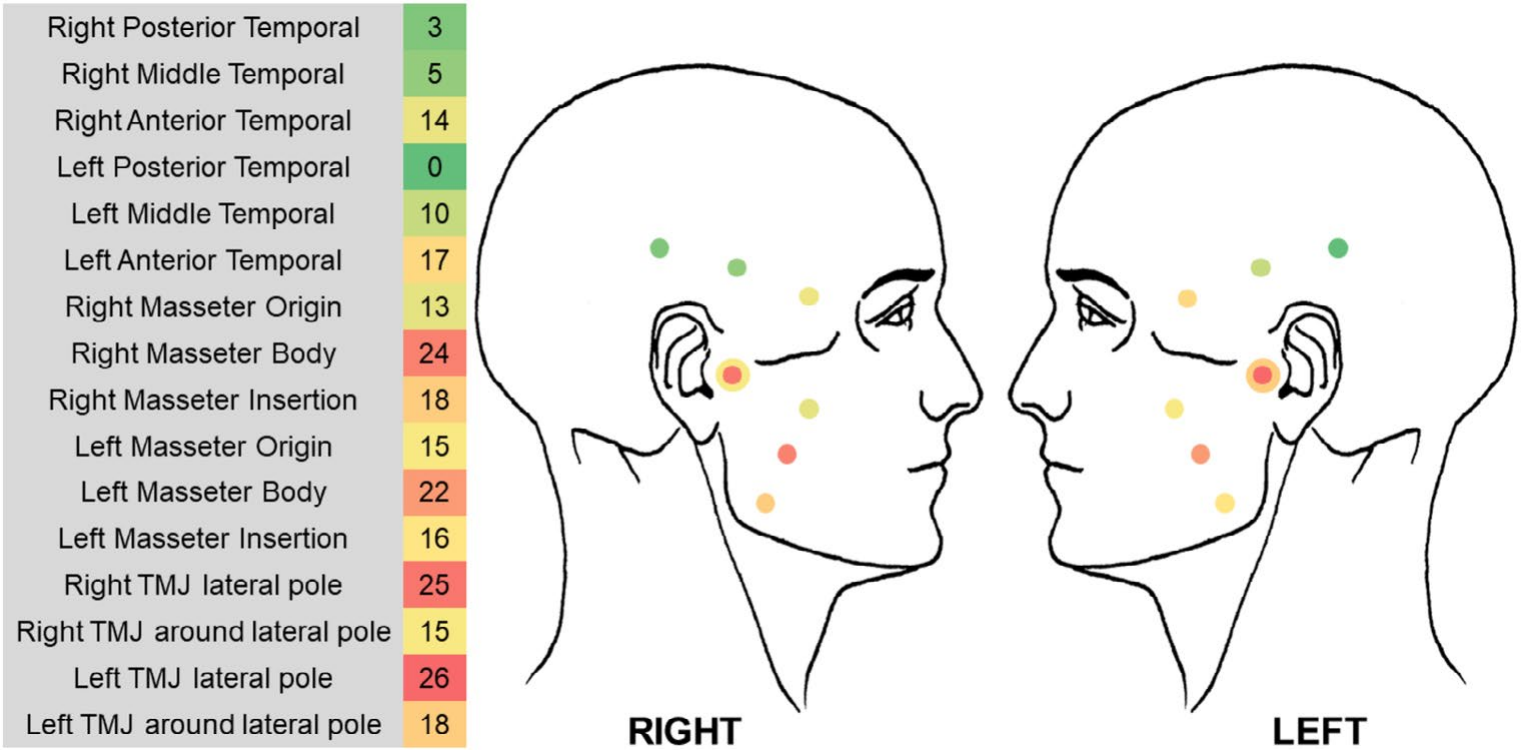
Representative heatmap of the prevalence of sites eliciting referred pain upon palpation in the total sample. The greenish colors represent lower prevalence, while the reddish colors indicate higher prevalence. The intensity of the colors further reflects the frequency of occurrence at each palpated site.

The median age of the sample was 38 years, with an interquartile range (25%–75%) of 34–50 years. The median sites eliciting pain upon palpation per individual were 2, with an interquartile range of 1–4. Figure 1 illustrates the muscular and articular sites as well as the frequency of referred pain upon palpation for each site in the sample.

### 3.2 Correlation

There was a statistically significant positive correlation only between the number of sites eliciting referred pain upon palpation and the clinical variables of pain frequency and chronic pain intensity (*p* < 0.001), as well as with the number of painful body sites (*p* = 0.009). No statistically significant correlation was observed for the variables of pain duration and pain‐related disability nor for any of the psychosocial or psychophysical variables (*p* > 0.05), as shown in Table 1.

**FIGURE 2:**
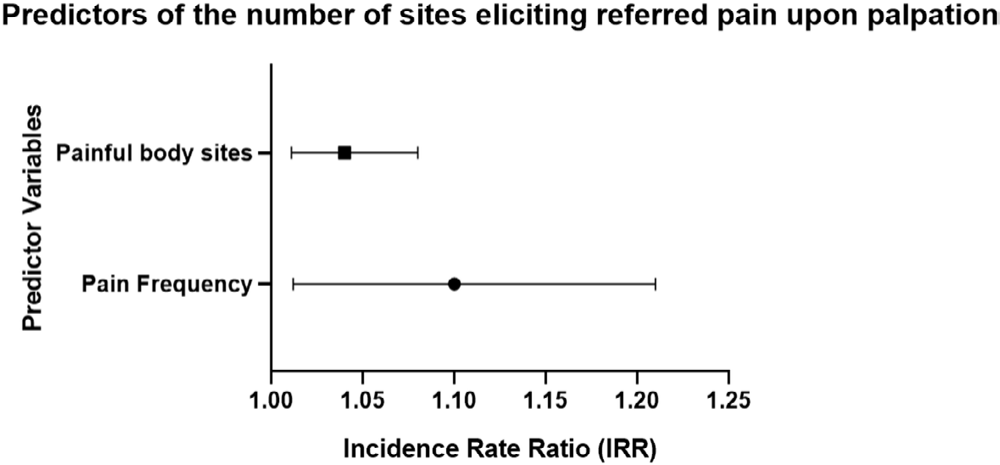
Forest plot illustrating the significant predictors of the number of sites eliciting referred pain upon palpation. The plot displays the rate ratios (RR) and 95% confidence intervals (CI) for the number of painful body sites and pain frequency, identified as significant predictors in the negative binomial regression model.

**TABLE 1:** Spearman's correlation results.

	Number of sites eliciting referred pain upon palpation	
	Rho de spearman	*p*
*Clinical features*		
Pain duration	0.223	0.051
Pain frequency	0.397	< 0.001*
Painful body sites	0.298	0.009*
Chronic pain intensity	0.390	< 0.001*
Pain‐related disability	0.146	0.206
*Psychossocial features*		
Stress (PSS)	−0.025	0.827
Anxiety (HADS)	−0.144	0.211
Depression (HADS)	0.007	0.951
Catastrofization (PCS)	−0.076	0.509
Sleep quality (PSQI)	0.039	0.736
*Psychophysical features*		
Trigeminal MPT	0.038	0.743
Extra‐Trigeminal MPT	−0.046	0.691
Trigeminal WUR	0.106	0.359
Extra‐Trigeminal WUR	−0.099	0.391
Trigeminal PPT	−0.117	0.312
Extra‐Trigeminal PPT	0.038	0.741
Trigeminal CPM	0.083	0.474
Extra‐Trigeminal CPM	0.139	0.227

*Note:* Spearman's correlation results. “*” Shows statistically significant values (*p* < 0.05).

Abbreviations: CPM, conditioned pain modulation; MPT, mechanical pain threshold; PPT, pressure pain threshold; WUR, wind‐up ratio.

### 3.3 Regression

Regression analysis using the negative binomial model identified the number of painful body sites and pain frequency as significant predictors of the number of sites eliciting referred pain upon palpation (Tables 2 and 3, and Figure 2). Specifically, each additional painful site on the body was associated with a 4% increase in the rate of sites eliciting referred pain upon palpation, and each additional day of pain in the past week corresponded to a 10% increase. In contrast, age and chronic pain intensity were not significantly associated with the outcome (*p* > 0.05).

**TABLE 2:** Loglikelihood ratio test.

	*χ* ^2^	df	*p*
Age	2.62	1	0.106
Frequency	4.92	1	**0.027** *
Painful body sites	6.94	1	**0.008** *
Intensity	2.73	1	0.099

*Note:* Likelihood ratio tests for the contribution of each predictor to the negative binomial regression model. The chi‐square (*χ* ^2^) values refer to the individual effect of each variable in explaining the number of sites eliciting referred pain upon palpation.

* Shows statistically significant values (*p* < 0.05).

**TABLE 3:** Predictors of the number of sites eliciting referred pain upon palpation.

Names	Estimate	95% Exp (*B*) confidence interval				*z*	*p*
		SE	Exp (*B*)	Lower	Upper		
(Intercept)	1.0157	0.08826	2.76	2.317	3.28	11.51	< 0.001
Age	0.0132	0.00813	1.01	0.997	1.03	1.63	0.104
Frequency	0.0991	0.04367	1.10	1.012	1.21	2.27	**0.023** *
Painful body sites	0.0423	0.01556	1.04	1.011	1.08	2.72	**0.006** *
Intensity	0.0101	0.00618	1.01	0.998	1.02	1.63	0.103

*Note:* Parameter estimates for the negative binomial regression model predicting the number of sites eliciting referred pain upon palpation. Exp (B) represents the rate ratio for each predictor. Confidence intervals (95% CI) and *p*‐values are presented for each variable.

* Shows statistically significant values.

## 4 Discussion

The present study aimed to evaluate the relationship between the increase in the number of sites eliciting referred pain upon palpation in masticatory muscles and TMJ and its relationship with the biopsychosocial context of TMD patients. Our findings corroborate an additional negative repercussion on clinical parameters related to pain. Moreover, the worsening of pain, both in intensity and frequency, and the spread of painful body sites are correlated with the increased manifestation of referred pain in the trigeminal region. In addition, the last two variables together proved to be predictors of this increase.

It is important to note that the present sample consisted exclusively of women. Although this approach allows for greater control over hormonal variability, it also limits the generalizability of the findings. Sex‐related differences may significantly influence pain perception, the distribution of referred pain, and psychosocial responses (Graven‐Nielsen 2006). Factors such as hormonal fluctuations and differences in central pain modulation mechanisms between men and women may contribute to diverse clinical profiles (Law et al. 2008).

Among the few studies published on this topic (Winocur‐Arias et al. 2022; Barjandi et al. 2021; Varun et al. 2023; Lovgren et al. 2022), all included the presence of referred pain through hierarchical diagnosis, as suggested by the DC/TMD. In this context, the presence of referred pain has been associated with more severe pain intensity (Winocur‐Arias et al. 2022) and widespread pain across the body (Varun et al. 2023), findings that reinforce the results of the present study. Additionally, significant associations have been observed with psychosocial factors such as anxiety (Barjandi et al. 2021; Varun et al. 2023), depression (Winocur‐Arias et al. 2022; Barjandi et al. 2021; Varun et al. 2023), stress (Barjandi et al. 2021) and insomnia (Barjandi et al. 2021), as well as a direct relationship with pain sensitivity, characterized by a reduced pain threshold (Hong et al. 1996; Varun et al. 2023). To the best of our knowledge, no study has investigated whether an increase in sites eliciting referred pain upon palpation exerts a relevant influence on the overall context of TMD patients.

In the psychosocial domain, the amplification of referred pain does not appear to be particularly related to its worsening. The lack of a proportional correspondence between these variables does not rule out the possibility that psychosocial impairment is indeed linked to the presence of referred pain, as observed in other studies (Winocur‐Arias et al. 2022; Barjandi et al. 2021; Varun et al. 2023). Possibly, this relationship may be indirect and/or multifactorial, rather than occurring in a unidimensional way.

Regarding clinical parameters, a greater manifestation of referred pain correlates with higher reports of pain intensity, short‐term symptom persistence, and increased pain complaints in other body areas. These findings are consistent with temporal and spatial summation phenomena—common features of central sensitization, which is recognized as a contributing mechanism in referred pain (Graven‐Nielsen 2006; Sago et al. 2023; Domenech‐Garcia et al. 2016). Facilitated nociceptive signaling and increased central nervous system excitability may lead to neuroplastic changes. These alterations could explain the spread of pain to multiple body regions (Fernandez‐de‐Las‐Penas et al. 2023; Graven‐Nielsen and Arendt‐Nielsen 2010), often accompanied by a higher number of painful diagnoses. They may also account for pain being perceived at sites distant from the actual nociceptive stimulus (Arendt‐Nielsen et al. 2000; O'Neill et al. 2007) during physical examination. This rationale supports the idea that both widespread pain and referred pain may originate from a shared underlying mechanism of central processing, yet manifest in distinct ways.

This interpretation may seem inconsistent with the lack of significant correlations found in our study between referred pain and psychophysical parameters. These measures—such as increased mechanical pain sensitivity and impaired endogenous pain inhibition—are commonly used to assess central sensitization. However, this discrepancy may be explained by a methodological detail: psychophysical assessments were performed exclusively on the masseter muscle, while referred pain was evaluated across six distinct anatomical sites within the trigeminal region. All tests were standardized and applied to the same location (centered over the masseter muscle body) for all participants. This may have influenced responses depending on whether that site was associated with familiar pain and/or referred pain in each individual. Evidence suggests that more sensitive subregions within a muscle, such as tendinous areas, are more likely to elicit referred pain (Gibson et al. 2006). Furthermore, both peripheral and central mechanisms contribute to referred sensations in a relatively balanced manner (Sago et al. 2023), suggesting that a more detailed and site‐specific evaluation may be necessary to clarify these associations.

Considering that increased pain frequency and the spread of painful body sites have been identified as significant predictors of the intensification of referred pain manifestation in the trigeminal region, a multidisciplinary approach to pain management involving professionals from different specialties can enhance the outcomes of treatment targeted at the orofacial region. Furthermore, reducing the pain frequency should be prioritized as a therapeutic goal, reinforcing initial guidelines and promoting adherence to treatment. This approach shows patients the importance of ongoing collaboration, aimed not only at symptom relief in the orofacial region but also for a comprehensive and lasting improvement in their overall well‐being. Longitudinal studies are essential to assess whether the long‐term control of these factors consistently contributes to the reduction of referred pain. If such fluctuation exists, it is essential to determine whether this reduction occurs independently of treatment or if it can be modulated according to the type of therapeutic intervention applied.

As it is also found in healthy individuals (Domenech‐Garcia et al. 2016; Masuda et al. 2018), the possibility that referred pain elicited upon palpation represents a normal pathophysiological response to intense nociceptive stimuli cannot be excluded, particularly in individuals with pre‐existing conditions of increased pain sensitivity and deficiency in the endogenous pain modulation system (Sago et al. 2023; Palsson et al. 2021). Furthermore, considering the hypothesis of an individual “predisposition”, this phenomenon may be exacerbated by recurrent pain episodes, involving temporal and spatial summation, and could potentially be understood as a consequence by the end of the process. To date, a cause‐and‐effect relationship between these components cannot be established.

Preliminary studies suggest that the use of antidepressants may enhance the modulation of the endogenous inhibitory system in the presence of referred pain (Haviv et al. 2015), and better outcomes have been observed with botulinum toxin use in patients without myofascial referred pain (Abboud et al. 2017). The influence of conservative treatments, psychological therapies, and pharmacological prescriptions should be tested either individually or in combination to assess their effect on the manifestation of referred pain upon palpation. This understanding will help determine whether the presence and/or increase in referred pain can be considered a reliable indicator for estimating the initial prognosis and guiding more targeted treatment, potentially improving patients' overall clinical outcomes.

The count of sites eliciting referred pain sensations during the physical examination is a simple and widely applicable tool in clinical practice, particularly useful for pain specialists in the diagnostic context, according to the DC/TMD criteria (Schiffman et al. 2014). The authors recommend the application of this methodology as a complement in assessing the severity of TMD, alongside data obtained from the patient history, while considering the relevance of pain on each patient's quality of life. This approach aims to improve the understanding of the condition's severity and its unique impact on each individual.

Among the limitations of this study, it should be noted that the sample included only women, which may restrict the generalizability of the results to other populations. Future studies including male participants are needed to better explore potential sex‐related differences in referred pain manifestations. Additionally, the psychophysical tests were performed exclusively on the masseter region, which may limit the conclusions regarding this parameter. The assessments were also conducted by a single examiner; however, standardized procedures and the diagnostic guidelines established by the DC/TMD were carefully followed, which may help minimize potential bias. Finally, this study is a cross‐sectional analysis based on secondary data; therefore, the results should not be extrapolated to the impact of increased referred pain manifestation on the prognosis and treatment of TMD.

## 5 Conclusion

The increase in referred pain manifestation upon palpation during the physical examination, as recommended by the DC/TMD, highlights a worsening in clinical pain‐related parameters, including its intensity, frequency, and spread of painful areas throughout the body. Clinicians should consider this tool as a complement in their patients' diagnosis, as it may contribute to a deeper understanding of the painful condition and its individual impact.

## 6 Perspective

This study highlights the significance of assessing referred pain sites in TMD diagnosis, showing their correlation with pain clinical severity. It identifies key predictors, such as pain frequency and widespread pain, offering clinicians a practical tool to better understand and manage the complexity of chronic pain in TMD patients.

## Author Contributions

**Beatriz Amaral de Lima‐Netto:** conceptualization, methodology, software, investigation, data curation, formal analysis, writing – original draft. **Paulo César Rodrigues Conti:** validation, visualization, funding acquisition, writing – review and editing, resources. **Rafaela Stocker Salbego:** methodology, formal analysis, investigation, data curation, writing – review and editing, visualization. **Matheus Herreira‐Ferreira:** methodology, formal analysis, investigation, data curation, visualization, writing – review and editing. **Yuri Martins Costa:** conceptualization, validation, visualization, resources, writing – review and editing, project administration. **Leonardo Rigoldi Bonjardim:** conceptualization, methodology, formal analysis, resources, writing – review and editing, funding acquisition, project administration.

## Ethics Statement

Ethical approval for this study was obtained from the Research Ethics Committee of the Bauru School of Dentistry at the University of São Paulo (CAAE 75050723.0.0000.5417), on 10/18/2023.

## Consent

This study involved adult volunteers of legal age.

## Conflicts of Interest

The authors declare no conflicts of interest.

## Data Availability

The data that support the findings of this study are available from the corresponding author upon reasonable request.
